# A four-gene signature identified by integrated transcriptomic analysis for differential diagnosis and prognosis of uterine smooth muscle tumors

**DOI:** 10.3389/fonc.2025.1591875

**Published:** 2025-08-04

**Authors:** Huiyi Hu, Yuanqun Chen, Bo Hong, Jia Liu, Yuchen Jiang, Zhiying Yu, Zhi-Jie Xiao, Jing Li

**Affiliations:** ^1^ Scientific Research Centre, The Seventh Affiliated Hospital, Sun Yat-sen University, Shenzhen, China; ^2^ Shenzhen Key Laboratory of Bone Tissue Repair and Translational Research, The Seventh Affiliated Hospital, Sun Yat-sen University, Shenzhen, China; ^3^ Department of Gynecology, Shenzhen Second People's Hospital, the First Affiliated Hospital of Shenzhen University, Shenzhen, China; ^4^ Shenzhen Key Laboratory of Reproductive Immunology for Peri-implantation, Shenzhen Zhongshan Institute for Reproductive Medicine and Genetics, Shenzhen Zhongshan Obstetrics & Gynecology Hospital, Shenzhen, China; ^5^ Department of Gynecology, Women's Hospital, School of Medicine, Zhejiang University, Hangzhou, China

**Keywords:** uterine smooth muscle tumors, transcriptome, biomarkers, early diagnosis, prognosis

## Abstract

**Introduction:**

Uterine leiomyomas (ULM) and uterine leiomyosarcomas (ULMS) are smooth muscle tumors of the uterus that share overlapping histopathological features but exhibit markedly different biological behaviors and clinical outcomes. Whereas ULMs are benign, ULMS are rare yet highly aggressive. Clinically, accurately distinguishing tumor tissue from normal myometrial tissue remains challenging, particularly due to substantial uncertainty in preoperative diagnosis. In this study, we aim to identify molecular biomarkers capable of distinguishing uterine smooth muscle tumors (including ULM and ULMS) from normal myometrium in order to uncover driver genes involved in tumorigenesis.

**Methods:**

We analyzed RNA-seq datasets from the Gene Expression Omnibus (GEO) and The Cancer Genome Atlas (TCGA) using GEO2R, Limma, and Weighted Gene Co-expression Network Analysis (WGCNA) to identify differentially expressed genes (DEGs) in ULMS and ULM.

**Results:**

Four hub genes—ABLIM1, FHL5, MAP3K8, and TOP2A—were consistently dysregulated in both tumor types relative to normal tissue, suggesting their common role in tumor development. Specifically, ABLIM1, FHL5, and MAP3K8 were downregulated, whereas TOP2A was upregulated, with further differential expression noted between ULMS and ULM. These findings were validated across independent cohorts and confirmed at the protein level via immunohistochemistry. Moreover, survival analysis revealed the prognostic significance of this four-gene signature: high TOP2A with low ABLIM1, FHL5, and MAP3K8 expression correlated with decreased overall survival in ULMS, implicating their potential role as diagnostic and prognostic markers.

**Discussion:**

Our study identifies ABLIM1, FHL5, MAP3K8, and TOP2A as key molecular drivers of uterine smooth muscle tumorigenesis. The four-gene signature shows promise as a biomarker panel for early diagnosis and differentiation between tumor and normal tissues, providing a potential molecular foundation for targeted therapeutic strategies.

## 1 Introduction

Uterine smooth muscle tumors include a variety of gynecological neoplasms, such as uterine leiomyomas (ULM) and uterine leiomyosarcomas (ULMS), which can range from benign to malignant. ULMs are quite common, affecting about 70-80% of women during their reproductive years, and they usually do not cause symptoms, appearing benign in nature (1). Although frequently asymptomatic, they can lead to a range of clinical problems, such as abnormal uterine bleeding, pelvic pain, and infertility (2). Conversely, ULMS, although rare, are known for their aggressive behavior and poor prognosis, representing about 3-7% of all uterine cancer cases, with a concerning 5-year survival rate of less than 50%, even when diagnosed early. The challenge of preoperative diagnosing ULMS is remain significant, particularly when compared to endometrial cancer, where an endometrial biopsy can successfully identify around 90% of affected individuals (3).

Given their common origin and histological similarity, uterine smooth muscle tumors pose diagnostic challenges when using traditional histopathological approaches, particularly in borderline lesions or early-stage tumors, where precise classification remains difficult. Conventional criteria such as mitotic counts, nuclear atypia, and necrosis, although effective in certain cases, are often subjective and exhibit low reproducibility in clinical practice (4–6). Hence, there is a growing need for reliable molecular or biochemical markers that can provide an objective and reproducible approach to distinguishing uterine smooth muscle tumors from normal myometrial tissue.

In addition to imaging and histological techniques used for preoperative assessment, serological markers such as CA-125 have been investigated. However, their sensitivity and specificity for detecting early-stage lesions remain limited, with significant elevations generally occurring only in advanced or progressive ULMS cases. Moreover, research conducted by Sagae et al. indicates that endometrial biopsies successfully diagnose uterine leiomyosarcoma in merely 35% of instances (7), underscoring the limitations of current diagnostic methods for early tumor detection. Thus, there is an urgent need to identify more sensitive and specific molecular or biochemical markers to enhance the accuracy and feasibility of preoperative diagnosis. Progress in the fields of molecular biology and genomics has facilitated the discovery of various promising biomarkers associated with tumor progression, regulation of the cell cycle, and apoptotic processes (8). Recent studies have begun to elucidate the genetic and molecular underpinnings of ULM and ULMS, revealing altered gene expression profiles and potential biomarkers that could improve diagnostic accuracy and therapeutic targeting. For example, MMP-2 and TEM1 have been found significantly upregulated in uterine tumors, and combined expression of MCM2 with p16 or Ki67 has shown potential discriminative capacity (9, 10).

Previous research has highlighted various biomarkers and pathways implicated in ULM and ULMS, yet systematic investigations into transcriptomic alterations between uterine smooth muscle tumors and normal myometrial tissues remain scarce, limiting our understanding of early molecular mechanisms underlying tumorigenesis. Although ULM and ULMS exhibit certain differences (11), significant dysregulation in numerous pathways compared to normal tissues indicates that these tumors may share a subset of oncogenic drivers. To explore the molecular landscape of ULM and ULMS, we employ an integrative bioinformatics approach to identify key genes consistently dysregulated in both tumor types relative to normal tissues. By integrating multiple datasets from the Gene Expression Omnibus (GEO) database, we utilize tools such as GEO2R, Limma, and Weighted Gene Co-expression Network Analysis (WGCNA) to identify molecular markers capable of distinguishing uterine tumor tissues from normal myometrial tissue, and to investigate their potential diagnostic and prognostic value.

## 2 Methods

### 2.1 ULM and ULMS data collection

To study the molecular genetic events in the development of myometrial tumors, we downloaded a set of RNA-SEQ gene expression matrices containing ULM and ULMS from Gene Expression Omnibus (GEO, http://www.ncbi.nlm.nih.gov/geo) as analysis datasets, including GSE36610, GSE64763, GSE68295, GSE95101, GSE119041 and GSE100338 (**Table 1**). In addition, we also downloaded RNA-SEQ data of 33 human cancers and matched normal samples from The Cancer Genome Atlas (TCGA, containing 33 cancer types) and Genotype Tissue Expression (GTEx, containing 54 normal tissues) datasets (**Table 1**). Given the scarcity of ULMS samples in public databases and the variability of tumor subtypes across datasets, we selected only four datasets (GSE36610, GSE64763, GSE68295, and GSE100338) with clearly defined tumor types and matched normal tissue for differential expression analysis and WGCNA, ensuring scientific rigor and analytical consistency. Other datasets, such as GSE95101, GSE119041, and TCGA-SARC, either lacked appropriate normal controls or predominantly contained non-ULMS subtypes—specifically, GSE119041 was composed of endometrial stromal sarcomas, while TCGA-SARC included uterine sarcomas samples without clearly specified subtypes. Including these datasets could introduce biological bias; thus, they were utilized solely as independent validation cohorts.

**Table 1:** Information of TCGA/GEO datasets containing the ULM and ULMS patients.

Dataset ID	Platform	Samples	Disease	Group
GSE36610	GPL7363	12 patients and 10 controls	ULMS	Discovery
GSE64763	GPL571	50 patients and 29 controls	ULM/ULMS	Discovery
GSE68295	GPL6480	6 patients and 3 controls	ULM/ULMS	Discovery
GSE100338	GPL21290	3 patients and 3 controls	ULM	Discovery
GSE95101	GPL13376	20 patients and 18 controls	ULM	Validation
GSE119041	GPL17692	50 patients and 0 controls	Uterine sarcoma	Validation
TCGA-SARC	GPL11154	29 patients and 2 controls	Uterine sarcoma	Validation

### 2.2 Identification of key genes in the occurrence of ULM and ULMS

Three bioinformatics tools (GEO2R, limma R, and WGCNA) were used to identify key genes in the occurrence of ULM and ULMS. The first method used GEO2R (https://www.ncbi.nlm.nih.gov/geo/geo2r/) to find DEGs. Genes that satisfied |log2(FC)|> 1 and p-value < 0.05 were statistically significant, and DEGs shared in all datasets from both diseases (identified in at least two IDC studies) were considered as key genes in the occurrence of ULM and ULMS in this method. Venn diagrams were used to show the overlap of DEGs between different element sets. The second method was to reorganize the gene expression matrices of ULM and ULMS from 4 GEO studies and eliminate batch effects to obtain 2 combined matrices. Co-expression modules in the combined matrices of ULM and ULMS were identified by weighted gene co-expression network analysis (WGCNA R package). WGCNA analysis was performed on 11701 genes using weighted gene co-expression network analysis (WGCNA R package) to generate co-expression modules in the combined matrices of ULM and ULMS. A soft threshold (R2 > 0.85) and a gene-gene correlation matrix were selected to construct an adjacency matrix to describe the degree of association between nodes. The adjacency matrix was then converted into a topological overlap matrix and a gene hierarchical clustering dendrogram was performed to identify co-expression modules. Finally, we calculated the module eigengenes (MEs) and the correlation between MEs and tissue properties to identify significantly differential modules. The overlap of DEGs in the above modules was represented by a Venn diagram. The third method was to perform differential gene analysis in the two combined matrices of ULM and ULMS using the limma R package. Satisfying |log2(FC)|> 1 and p-value < 0.05 was considered statistically significant. The overlap of DEGs in the above cohorts was represented by a Venn diagram.

### 2.3 Interaction network, functional enrichment analysis, and survival analysis of DEGs

The protein information within the protein-protein interaction (PPI) network of related DEGs was collected from the STRING database [https://string-db.org/]. Cytoscape tool version 3.9 and its Cytohubba plugin were used to filter and visualize hub genes according to the betweenness centrality scores in the PPI network. Gene Ontology (GO) and Kyoto Encyclopedia of Genes and Genomes (KEGG) enrichment analysis of differentially expressed genes (DEGs) or co-expressed genes was performed using KOBAS smart (KOBAS-i) version 3.0 [http://bioinfo.org/kobas/]. In addition, for survival analysis, we downloaded the survival data of TCGA-SARC and GEO119041 datasets from TCGA and GEO databases, respectively. The ggrisk package was called to construct a 4-gene (ABLIM1, FHL5, MAP3K8, and TOP2A) risk model, and patients were divided into high-risk and low-risk groups according to the risk scores generated by the system, and the relationship of the 4 genes combined on patient survival was evaluated. Data analysis and visualization were performed using GraphPad Prism software version 8.0 and [http://www.bioinformatics.com.cn/] bioinformatics tools. The relevant data and statistical analysis (p-values) of the results were automatically generated by the software and platform used.

### 2.4 Immunohistochemical staining (IHC)

Normal myometrium, fibroids, and leiomyosarcoma tissues were fixed with 4% paraformaldehyde in phosphate-buffered saline (PBS) and embedded in paraffin. Paraffin-embedded sections were subjected to IHC and hematoxylin and eosin (H&E) staining. All tissue sections underwent dewaxing, antigen fixation, blocking, and primary antibody incubation, including ABLIM1 (1:200, Proteintech, 15129-1-AP, China), FHL5 (1:100, Proteintech, 51015-1-AP, China), TOP2A (1:200, Proteintech, 20233-1-AP, China), and MAP3K8 (1:200, Abcam, ab196751, China). Subsequently, they were incubated with secondary antibodies and stained with DAB kit (P0203, Beyotime, China) for colour rendering and haematoxylin re-staining. Stained sections were imaged using a Nikon Eclipse Ni-U microscope (Tokyo, Japan).

## 3 Results

### 3.1 Identification of shared differentially expressed genes between uterine smooth muscle tumors and normal tissues using three bioinformatics methods

**Figure 1:**
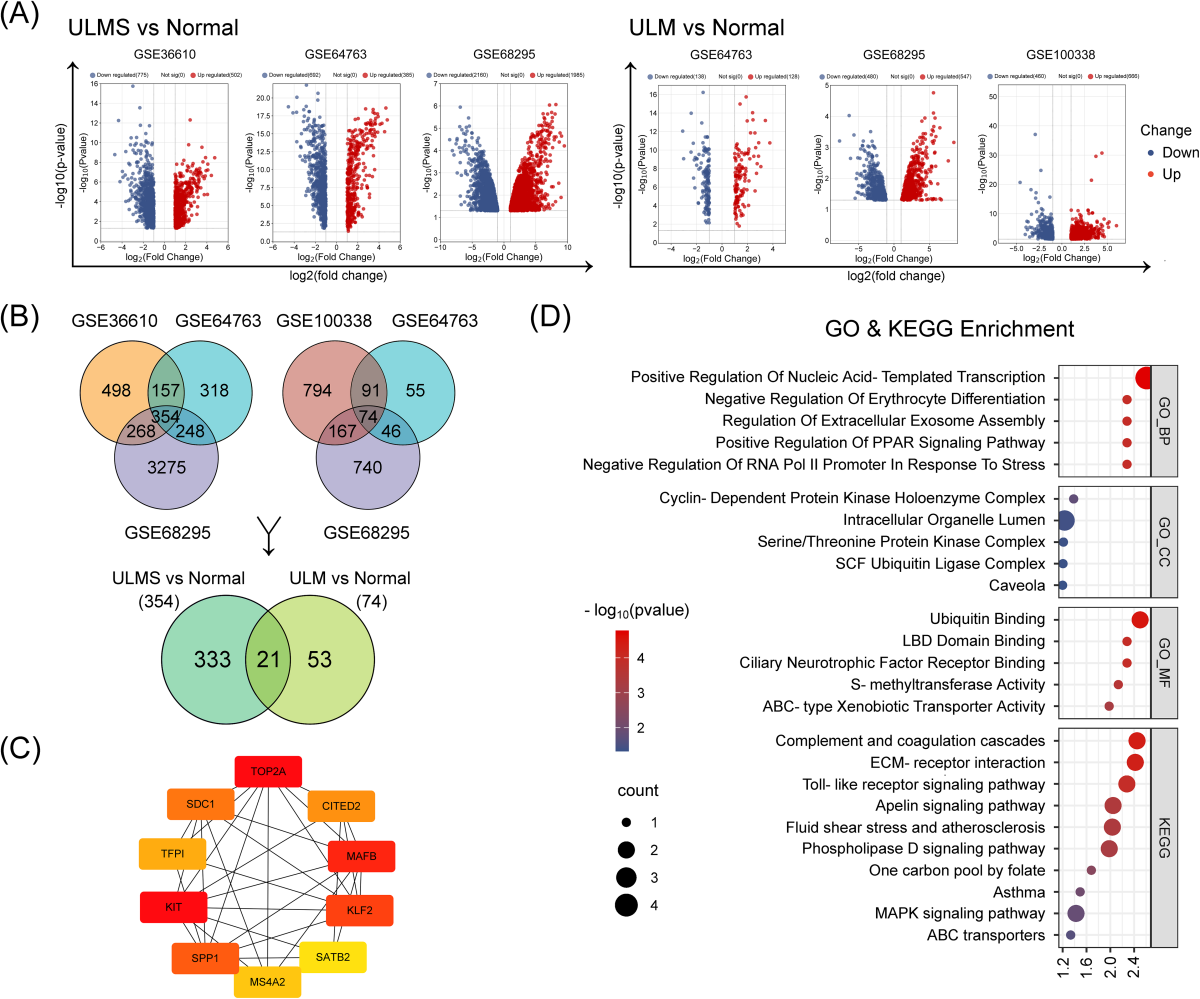
Identification of differentially expressed genes (DEGs) from uterine leiomyoma (ULM) and uterine leiomyosarcoma (ULMS) transcriptomic datasets using GEO2R. **(A)** The volcano plots illustrate differential gene expression across three ULM and three ULMS datasets. Red and blue dots represent significantly upregulated and downregulated genes, respectively, with the y-axis showing –log10 (p-value) and the x-axis indicating log2 (fold change). **(B)** The Venn diagrams reveal the intersection of DEGs across different datasets. The two upper Venn diagrams show overlapping DEGs within ULMS vs. Normal (left) and ULM vs. Normal (right). The lower Venn diagram indicates 21 overlapping genes shared between these two groups of DEGs. **(C)** The protein–protein interaction (PPI) network displays the interplay among the top 10 hub DEGs ranked by BC (betweenness centrality) scores, as determined by the Cytoscape Cytohubba plugin. The color scale from red to yellow corresponds to high to low BC values. **(D)** GO and KEGG enrichment analyses present the functional annotations of the 21 DEGs.

To explore shared molecular features that are commonly and significantly altered in uterine smooth muscle tumors (including ULM and ULMS) relative to normal uterine tissue, we integrated three complementary bioinformatics methods, including GEO2R, Limma, and weighted gene co-expression network analysis (WGCNA), to screen for hub genes or differentially expressed genes (DEGs) that might be critical the pathogenesis of both ULM and ULMS. First, we used GEO2R to analyze DEGs in multiple independent RNA-seq datasets (GSE36610, GSE64763, GSE68295, and GSE100338) from the GEO database, defining |log2(FC)| > 1 and p < 0.05 as the screening threshold. Compared with normal tissues, 354 genes were significantly dysregulated in ULMS, whereas only 74 genes showed differential expression in ULM (**Figures 1A, B**). A total of 21 DEGs were found to be shared by ULM and ULMS (**Figure 1B**), representing potential key regulators involved in the transition from normal myometrium to tumor. Protein-protein interaction (PPI) network analysis via STRING revealed a core regulatory circuit orchestrated by key transcription factors (TOP2A, KIT, MAFB, and KLF2; **Figure 1C**). Gene Ontology (GO) enrichment highlighted dysregulated biological processes, including nucleic acid-templated transcription, erythrocyte differentiation, and extracellular exosome assembly, with prominent molecular features in ubiquitin binding and cyclin-dependent kinase complexes. Moreover, KEGG pathway analysis highlighted perturbations in the complement cascade, ECM–receptor interactions, and Toll-like receptor signaling, offering novel insights into shared pathogenic mechanisms and potential therapeutic targets of ULMS and ULM (**Figure 1D**).

**Figure 2:**
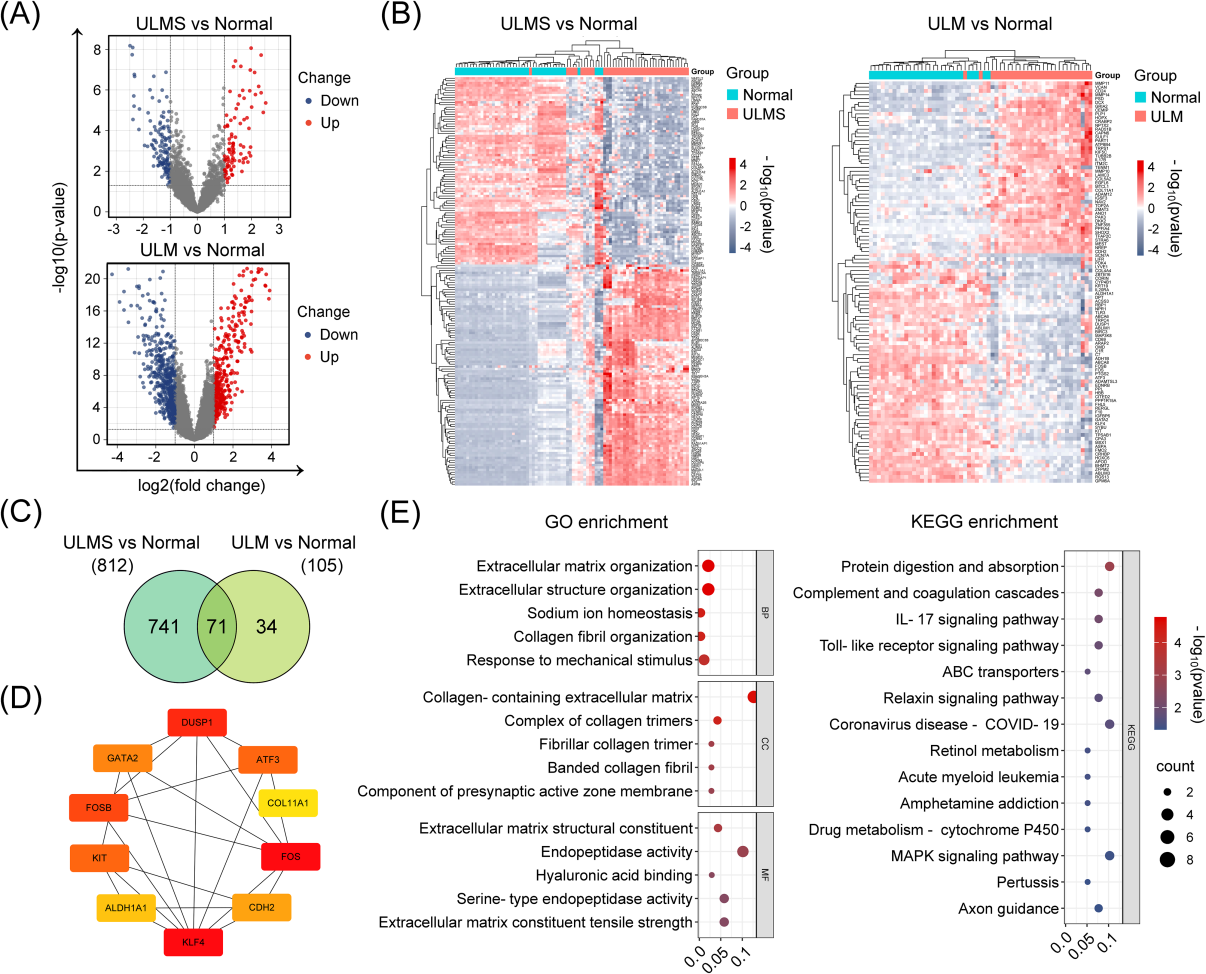
Identification and analysis of DEGs in ULMS and ULM datasets using the limma R package. **(A)** The volcano plots show the distribution of DEGs in ULMS and ULM after batch effect removal and data integration. Red and blue dots represent significantly upregulated and downregulated genes, respectively, with the y-axis indicating –log10 (p-value) and the x-axis indicating log2 (fold change). **(B)** An unsupervised hierarchical clustering heatmap highlights the marked transcriptomic differences between ULMS/ULM and normal tissues. The color gradient from red (upregulated) to blue (downregulated) graphically depicts changes in gene expression levels. Sample clustering presents distinct disease-specific groupings. **(C)** The Venn diagram analysis shows the DEGs identified in the ULMS and ULM groups and the number of overlapping genes. **(D)** The PPI network illustrates interactions among the top 10 key genes (among 71 overlapping DEGs) ranked by their BC (betweenness centrality) scores via the CytoHubba plugin in Cytoscape. Colors range from red to yellow to reflect high to low BC values. **(E)** GO and KEGG enrichment analyses reveal the functional annotations of the 71 DEGs.

In addition, we performed an integrated analysis of five datasets to further characterize the molecular differences between uterine tumor tissues and normal myometrium. After batch-effect correction and differential analysis with the Limma R package (|log2(FC)| > 1, p < 0.05 as thresholds) of the integrated ULM and ULMS expression matrices, we identified 812 dysregulated genes in ULMS and 105 dysregulated genes in ULM. Among them, 71 genes exhibited consistent aberrant expression across both tumor types (**Figures 2A,C**), suggesting a set of molecular signals that may represent stable alterations during the transition of uterine smooth muscle cells from normal to tumor states. Unsupervised hierarchical clustering revealed a clear transcriptomic distinction between tumor samples and normal tissues (**Figure 2B**). String analysis of the protein–protein interaction network then identified a core regulatory module centered on key transcription factors including DUSP1, GATA2, KIT, and ATF3 (**Figure 2D**), suggesting their potential roles in orchestrating uterine tumorigenesis. Functional enrichment analysis revealed significant perturbations in extracellular matrix organization, collagen fibril assembly, and responses to mechanical stimulus (**Figure 2E**). Additionally, KEGG pathway analysis underscored dysregulation in protein digestion, the complement cascade, and IL-17 signaling, collectively shedding light on the unique molecular mechanisms driving these uterine smooth muscle tumors (**Figure 2E**).

**Figure 3:**
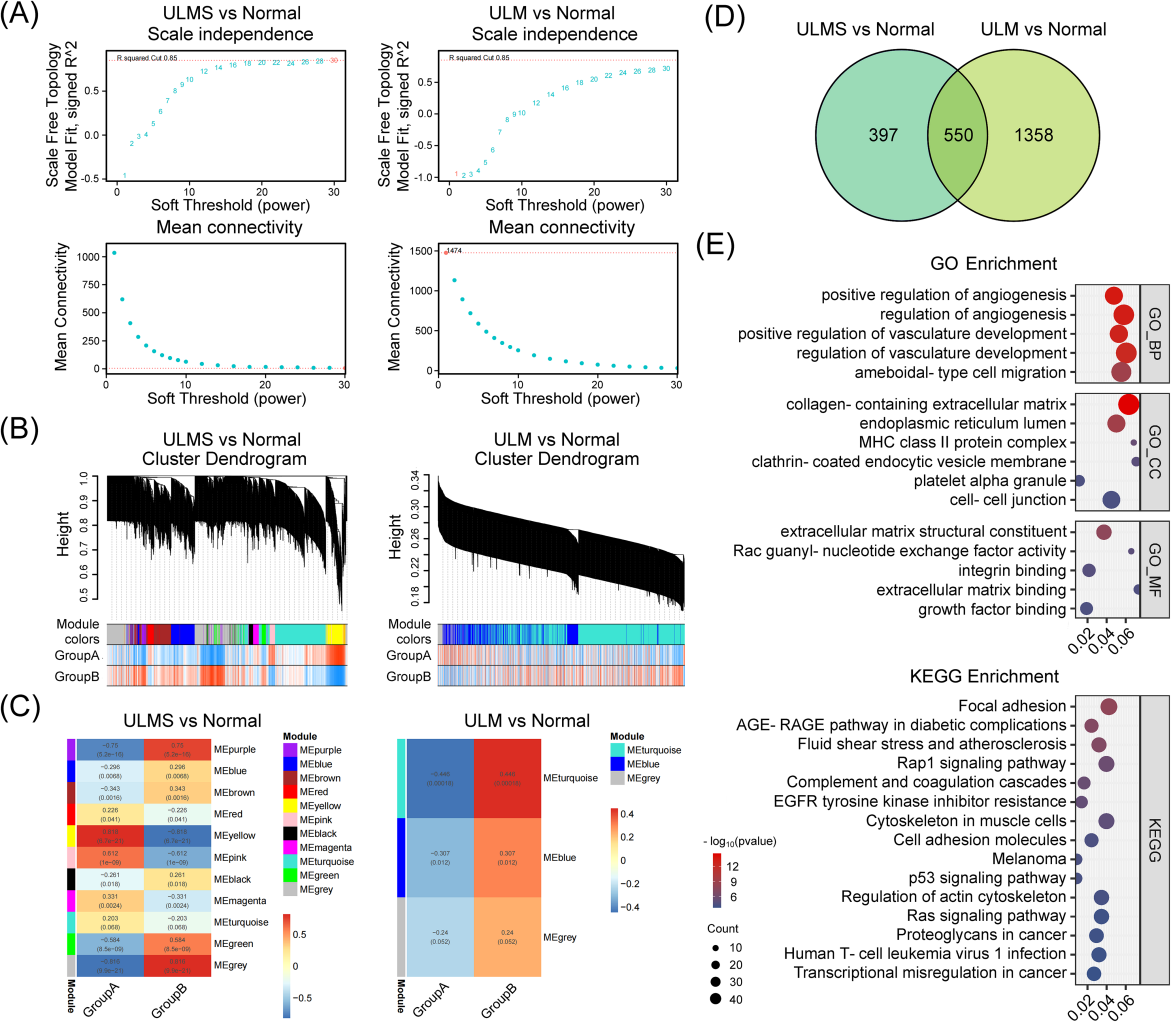
Weighted gene co-expression network analysis (WGCNA) identifies and characterizes hub genes in ULMS and ULM datasets. **(A)** Scale independence and mean connectivity analyses comparing ULMS and ULM with normal tissues. Both datasets reveal appropriate soft threshold choices, ensuring reliable network construction. **(B)** The cluster dendrogram shows the partitioning of gene modules. Using a dynamic tree-cutting algorithm, ULMS and ULM datasets were separately grouped into distinct co-expression modules, each represented by a different color. The heatmap below illustrates the sample distribution in Group A and Group B. **(C)** The module–trait correlation heatmap indicates the strength of association between modules and specific phenotypic traits. In the ULMS group, several modules display significant positive or negative correlations, whereas the ULM group exhibits a relatively simpler correlation pattern. **(D)** The Venn diagram displays the number of DEGs and their overlap in the ULMS and ULM groups. **(E)** GO and KEGG enrichment analyses reveal the functional annotations of the 550 shared hub genes.

Finally, to further investigate gene modules exhibiting coordinated expression changes between tumor and normal tissues, we conducted weighted gene co-expression network analysis (WGCNA) on the integrated ULMS and ULM expression matrices. Optimizing via soft-thresholding topology analysis revealed the presence of multiple highly organized and functionally relevant co-expression modules in uterine tumor tissues. ULMS showed complex regulatory patterns characterized by multiple significantly correlated modules, while ULM exhibited a more streamlined associational structure (**Figures 3A–C**). Specifically, significant differential modules included MEturquoise and MEbrown in ULM, and MEpurple, MEblue, MEbrown, MEred, MEyellow, MEpink, MEblack, MEmagenta, MEgreen, and MEgrey in ULMS (p < 0.05). Differential expression analysis pinpointed 947 ULMS-specific genes and 1,908 ULM-specific genes, with 550 hub genes shared between the two groups (**Figure 3D**). These shared genes may represent a fundamental regulatory network underlying the transition of uterine smooth muscle cells from normal to tumor states. Subsequent GO enrichment analyses revealed significant dysregulation of biological processes such as regulation of angiogenesis, blood vessel development, and extracellular matrix organization. KEGG Pathway analyses demonstrated disruptions in focal adhesion, the AGE–RAGE signaling cascade, and cytoskeleton regulation, collectively offering insights into the distinct molecular mechanisms underlying ULMS and ULM (**Figure 3E**). Collectively, these findings highlight a set of core co-expression networks potentially involved in the transformation of normal uterine tissue into tumor tissue, providing a molecular foundation for the identification of diagnostic or therapeutic targets.

### 3.2 ABLIM1, FHL5, MAP3K8, and TOP2A are consistently dysregulated core genes in uterine smooth muscle tumors compared to normal tissue

**Figure 4:**
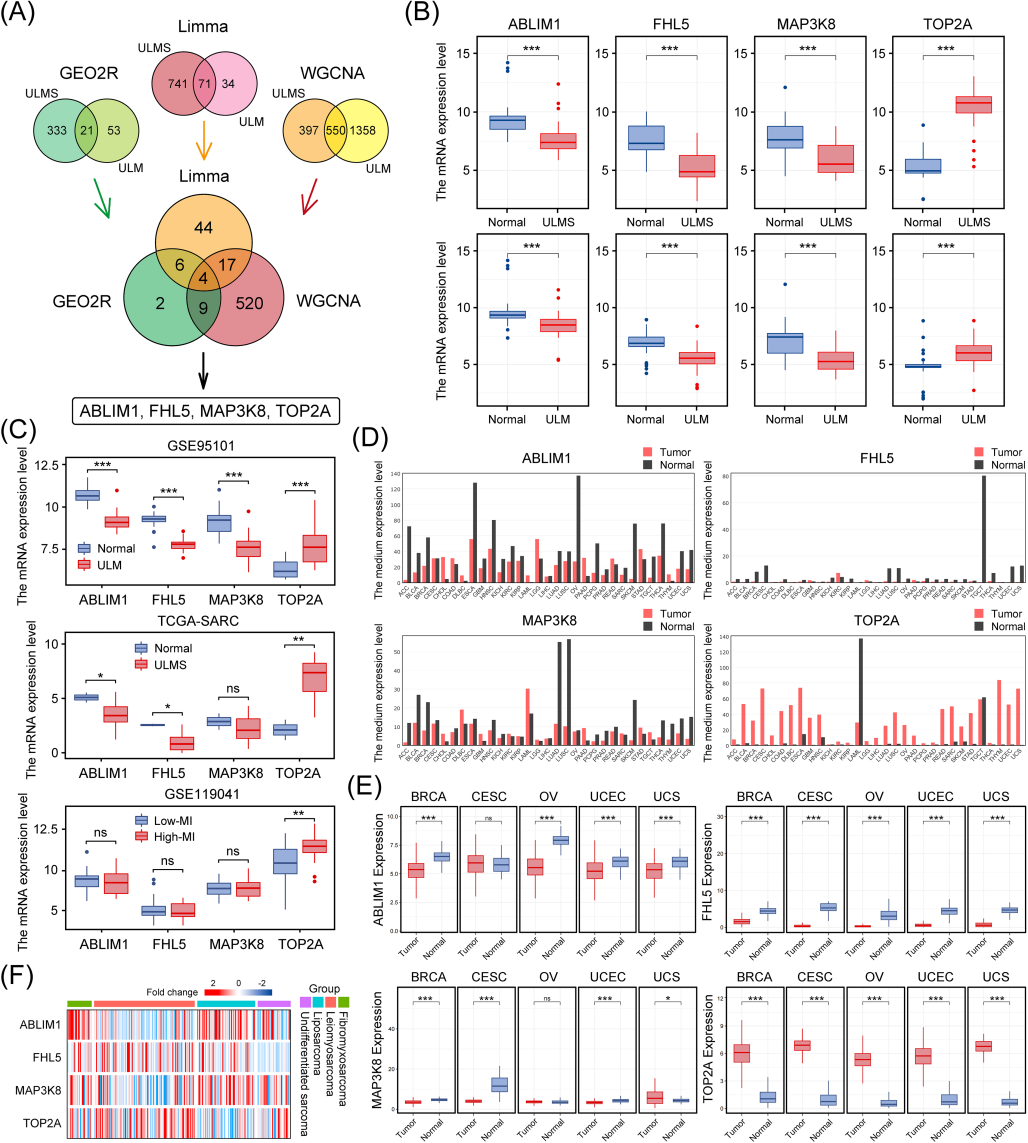
Identification of key hub genes in ULMS and ULM datasets using three bioinformatics approaches, and their expression patterns in multiple cancer types. **(A)** The Venn diagram illustrates the integration of three distinct methods, resulting in the identification of four key genes (ABLIM1, FHL5, MAP3K8, and TOP2A). **(B)** The mRNA levels of ABLIM1, FHL5, MAP3K8, and TOP2A in the ULMS and ULM datasets used in this study. **(C)** Three independent cohorts (GSE95101, TCGA-SARC, and GSE119041) validated the differential expression of these genes. **(D)** Bar plots from the GEPIA database show the expression profiles of ABLIM1, FHL5, MAP3K8, and TOP2A in 33 types of malignant tumors paired with corresponding normal tissues. **(E)** The expression patterns of the four genes in five female cancer types (BRCA, CESC, OV, UCEC, and UCS). **(F)** A heatmap compares the expression levels of the four genes in different sarcoma samples from the SARC dataset in the TCGA database. The color gradient indicates changes in expression levels. *p < 0.05, **p < 0.01, ***p < 0.001; ns, not significant.

To uncover common driver genes in ULM and ULMS that might guide early diagnosis and intervention, we integrated the results of three complementary bioinformatics approaches (GEO2R, the Limma R package, and WGCNA). This led us to a robust hub gene set comprising 4 genes: ABLIM1, FHL5, MAP3K8, and TOP2A. These genes are potentially involved in the tumorigenic processes of uterine smooth muscle tumors (ULM and ULMS) (**Figure 4A**; **Additional File 1: Figure 1**). Further examination of the integrated dataset in this study revealed that these four genes were differentially expressed in both tumors (p < 0.001), with ABLIM1, FHL5, and MAP3K8 showing a significant decrease in expression, whereas TOP2A expression was notably increased, compared to normal tissue (**Figure 4B**).

We then further validated our findings in multiple independent cohorts. In both the GSE95101 (ULM) and TCGA-SARC (uterine sarcoma) cohorts, these genes exhibited consistent expression trends, indicating a relatively uniform expression pattern across different uterine tumor subtypes (**Figure 4C**). Additionally, using dataset GSE119041 for ULMS, it was found that in ULMS patients with high mitotic indices, TOP2A expression showed a stronger correlation with mitotic index, suggesting a role in proliferative activity (**Figure 4C**). Consistent differential expression trends for these genes were also observed in a variety of other malignancies as well (**Figure 4D**). To establish broader clinical relevance, we extended our analyses to major gynecological malignancies, including breast cancer (BRCA), cervical cancer (CESC), ovarian cancer (OV), uterine endometrial carcinoma (UCEC), and uterine carcinosarcoma (UCS). This pan-gynecological cancer analysis revealed marked and consistent differential expression patterns (p < 0.001) (**Figure 4E**), suggesting a conserved expression pattern in diverse oncogenic contexts. Hierarchical clustering further validated that this gene set effectively distinguished uterine tumor samples from other sarcoma subtypes, supporting its potential diagnostic utility (**Figure 4F**). The consistency and reproducibility of these results across multiple independent datasets and platforms underscores the robustness and potential clinical diagnostic significance of this hub gene set.

### 3.3 Potential clinical application of the four-gene signature in uterine tumor identification and prognosis prediction

**Figure 5:**
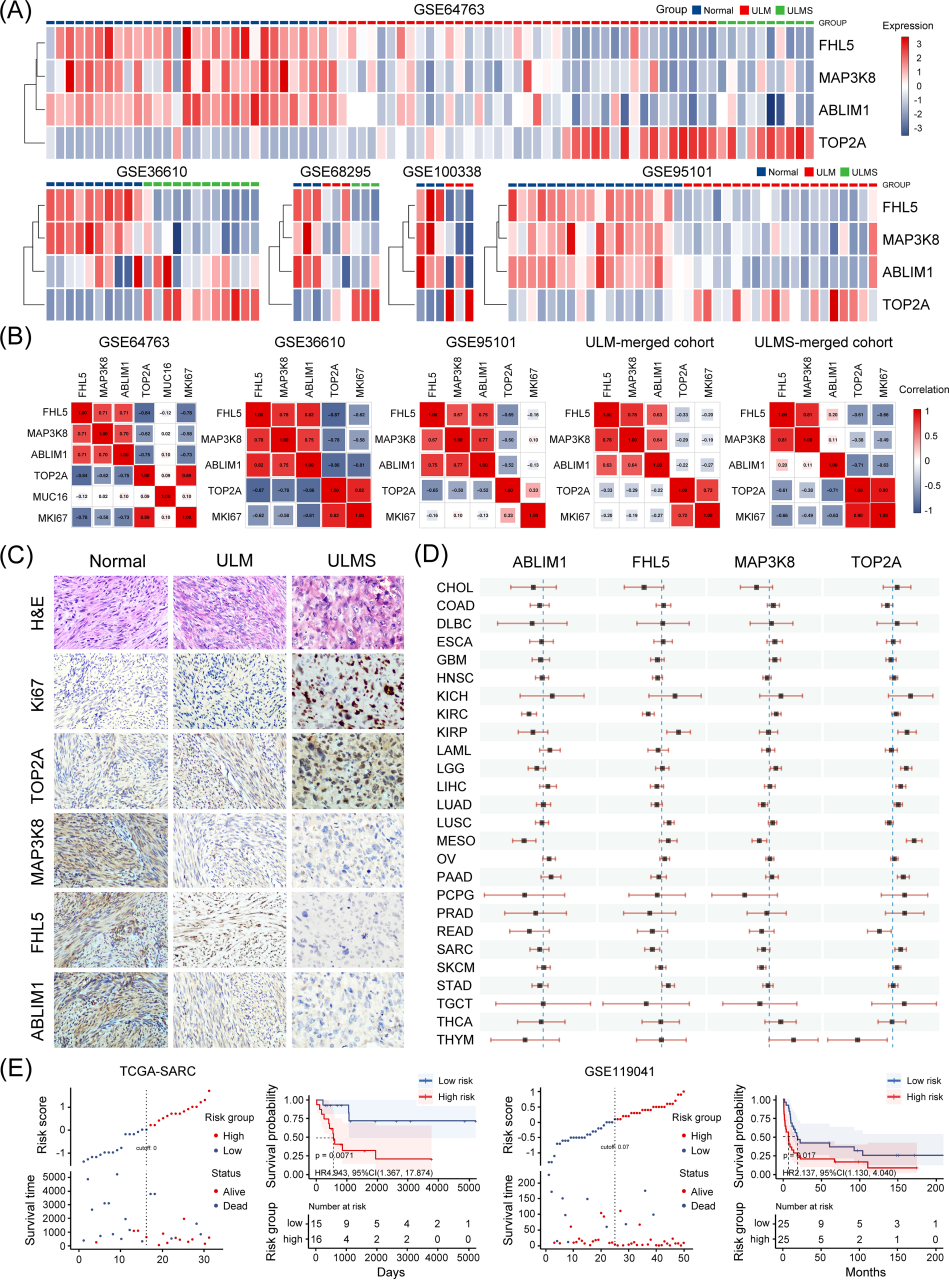
Clinical significance of the four candidate genes. **(A)** The heatmap showing that the four-gene expression signature effectively distinguishes uterine tumor samples (ULM and ULMS) from normal myometrial tissues across multiple GEO dataset samples in this study. The color gradient from blue to red indicates low to high expression, respectively. **(B)** Correlation analysis between the expression levels of the four candidate genes and known biomarkers such as CA-125 (encoded by MUC16) and Ki-67 (encoded by MKI67) across multiple datasets included in this study. MUC16 expression data was only available in the GSE64763 dataset. **(C)** Representative IHC staining images of the tested markers in normal myometrium, ULM, and ULMS tissues (magnification ×200). Four samples per group were stained, and representative images were selected for display. **(D)** A simplified forest plot displaying the results of univariate Cox analyses for the four genes across 33 types of malignancies. Each horizontal line corresponds to a hazard ratio with its 95% confidence interval indicated by the line’s endpoints. **(E)** A four-gene risk model was constructed using the ggrisk package for sarcoma samples in the TCGA-SARC and GSE119041 datasets. Patients were stratified into high-risk and low-risk groups based on their risk scores, and survival curves were generated accordingly.

In both the discovery (GSE36610, GSE64763, GSE68295, and GSE100338) and validation (GSE95101) cohorts, hierarchical clustering based on the four-gene set consistently distinguished uterine tumor samples from normal tissues. The clustering results revealed distinct transcriptomic profiles between tumor and non-tumor tissues, highlighting the discriminatory power of this gene set (**Figure 5A**). Correlation matrix analysis further showed that ABLIM1, FHL5, and MAP3K8 exhibited strong positive correlations with each other, while their expression levels were negatively correlated with those of TOP2A and MKI67 (encoding the canonical proliferation marker Ki-67) (**Figure 5B**). To further delineate weather the differential expression of this gene set could facilitate the differential diagnosis between ULM and ULMS, we subsequently analyzed the expression of the four genes by immunohistochemistry (IHC) to verify the alternation at protein level, expression of Ki-67 was used as a positive control. Representative IHC staining images of these markers in various tissues are shown in **Figure 5C**. Ki-67 staining patterns were consistent with previous findings, confirming the reliability and comparability of IHC assessments. In line with mRNA-level changes, protein levels of ABLIM1, FHL5, and MAP3K8 decreased progressively from normal tissue to tumor tissues, whereas TOP2A showed an opposite trend (**Figure 5C**). Moreover, we also analyzed the prognostic value of the four genes in different types of tumors (**Figure 5D**). In kidney renal clear cell carcinoma (KIRC) and Mesothelioma (MESO), high expression of ABLIM1 was significantly associated with better prognosis. In CHOL, HNSC, SARC, and LUAD, high expression of FHL5 and MAP3K8 seemed to be associated with better prognosis. In contrast, high expression of TOP2A was significantly associated with worse prognosis in KIRP, LGG, LIHC, LUAD, MESO, PAAD, and SARC. To elucidate the potential relationship between the four candidate genes and tumor prognosis, we further examined the combined prognostic value of these genes in two uterine sarcoma cohorts. Using the ggrisk package, we constructed a multivariate Cox regression model based on the four-gene set and stratified patients into high-risk and low-risk groups based on their risk scores (automatically generated by the system) in order to determine how the expression of these driver genes affects patient outcomes in different uterine sarcoma cohorts. In the TCGA-SARC ULMS cohort (n = 31), high-risk patients (exhibited higher TOP2A levels and lower levels of ABLIM1, FHL5, and MAP3K8 compared with the low-risk group) had a worse overall survival (p = 0.0071; HR = 4.943, 95% CI: 1.367–17.874) (**Figure 5E**). Consistently, a similar trend was observed in another independent uterine sarcoma cohort (GSE119041, n = 50) (p = 0.017; HR = 2.137, 95% CI: 1.130–4.040) (**Figure 5E**). These results suggest that the four-gene signature not only facilitates the identification of uterine tumor tissues but also serves as a valuable prognostic biomarker, potentially guiding clinical risk stratification and therapeutic decision-making.

## 4 Discussion

Despite the histological and imaging similarities between uterine leiomyomas (ULM) and uterine leiomyosarcomas (ULMS), identifying their distinct and shared molecular features remains critical for the early diagnosis of uterine tumors. Current clinical approaches often lack sufficient sensitivity for preoperative tumor identification, particularly in detecting early molecular alterations that characterize the transition from normal to neoplastic states. Therefore, defining a molecular signature that is broadly applicable to uterine smooth muscle tumors and capable of reliably distinguishing tumor tissue from normal myometrium holds significant clinical value. Although recent integrative genomic analyses have implicated recurrent mutations in TP53, ATRX, PTEN, and MEN1, as well as homologous recombination deficiency (HRD), in the pathogenesis of ULMS (12–14), transcriptome-wide integrative studies—especially those aimed at uncovering shared tumorigenic mechanisms rather than subtype-specific alterations—remain limited.

In this study, we conducted a thorough transcriptomic investigation to comprehensively characterize transcriptomic differences between uterine smooth muscle tumors (ULM and ULMS) and normal uterine tissue using multiple bioinformatics approaches, including GEO2R, Limma, and Weighted Gene Co-expression Network Analysis (WGCNA). Our analyses unveiled four hub genes—ABLIM1, FHL5, MAP3K8, and TOP2A—that consistently emerged as differentially expressed across all tumor samples when compared to normal tissue. Specifically, ABLIM1, FHL5, and MAP3K8 were downregulated, while TOP2A was upregulated. This four-gene signature demonstrated high stability across multiple independent datasets and was further validated at the protein level by immunohistochemistry. Notably, a clustering model based on the expression profiles of these genes reliably separated tumor tissues from normal controls, underscoring their potential as a unified biomarker panel for early tumor identification. These findings not only offer critical insights into the shared molecular mechanisms underlying uterine tumorigenesis but also provide a foundational framework for clinical molecular classification and prognostic assessment.

Recently, several studies have investigated the transcriptomic profiling of ULMS, aiming at identifying biomarkers for ULMS diagnosis and prognosis prediction (15–17). Integrated transcriptomic analysis on ULMS and ULM performed by Machado-Lopez et al. reported a set of 19 genes panel was dysregulated in ULMS compared to ULM, highlighting the role of cell cycle regulation, extracellular matrix remodeling, and cytokine signaling in tumor progression, which can be used as molecular signature for differential diagnosis of ULMS and ULM (16). However, none of the genes in this 19-gene set were overlapped with the 4-gene set we reported here, highlighting the complexity of ULMS biology and the importance of integrating multiple datasets and analytical approaches. Meanwhile, this discrepancy further emphasizes the importance of delineating the shared featured of ULM and ULMS. By focusing on genes dysregulated in both ULM and ULMS, we identified a core signature that may be instrumental for early diagnosis. This signature is dysregulated in both ULM and ULMS compared to normal tissues, and further differentially expressed in ULMS compared to ULM. Furthermore, we demonstrated that the expression profile of these four genes correlates with patient outcomes, suggesting that ABLIM1, FHL5, MAP3K8, and TOP2A not only mark structural differences but may also drive disease aggressiveness.

Among these four genes, TOP2A has been extensively studied in various malignancies as a prognostic marker and potential therapeutic target. TOP2A is a crucial enzyme involved in DNA replication and cell cycle progression. It is responsible for managing DNA topology by creating transient double-strand breaks. Its role in these fundamental cellular processes makes it a target for various anticancer therapies, particularly those involving chemotherapeutic agents that stabilize the TOP2A-DNA cleavage complex, leading to cell death (18). TOP2A has been reported to be a prognostic marker for various cancers. Overexpression of TOP2A has been reported to predict poor survival in breast cancer, prostate cancer, hepatocellular carcinoma and colorectal cancer (19–22). Besides, TOP2A has also been reported to be a marker for predicting drug response. Co-amplification of TOP2A and HER2 genes in breast cancer is associated with improved outcomes when treated with anthracyclines, highlighting the enzyme’s role as a predictive biomarker for chemotherapy efficacy (23). The involvement of TOP2A in cancer is not limited to its role as a biomarker. It also plays a direct role in tumor progression. In gallbladder cancer, TOP2A promotes cell proliferation and metastasis through the activation of the PI3K/Akt/mTOR signaling pathway, suggesting that it could serve as a therapeutic target (24). TOP2A has also been reported to involve in resistance to regorafenib, a common treatment for hepatocellular carcinoma (HCC). Silencing TOP2A has been shown to enhance HCC cells’ sensitivity to regorafenib, suggesting that targeting TOP2A may be a promising therapeutic strategy to alleviate resistance and improve treatment efficacy (25). We observed that TOP2A is consistently upregulated across all uterine tumor samples, with its expression levels positively correlated with mitotic activity, suggesting a potential driver role in the formation and progression of uterine tumors. This expression pattern provides strong support for TOP2A as both a diagnostic and prognostic biomarker, with promising potential for inclusion in future clinical assessment frameworks.

ABLIM1, FHL5, and MAP3K8, on the other hand, were downregulated in ULMS, indicating their potential tumor-suppressive roles. In contrast to TOP2A, the remaining three genes—ABLIM1, FHL5, and MAP3K8—exhibit more context-dependent roles in cancers. ABLIM1 is known for its role in cytoskeletal organization and developmental pathway. The role of ABLIM1 in cancer is controversial and cancer type dependent. In colorectal cancer (CRC), ABLIM1 has been identified as an oncogenic factor, promoting tumor growth and metastasis through the activation of the NF-ĸB signaling pathway. This is achieved by targeting IĸBα ubiquitination, which underscores its potential as a therapeutic target in CRC (26). In hepatocellular carcinoma (HCC), ABLIM1 is involved in actin polymerization and cell migration, processes crucial for cancer metastasis. Rictor, a component of the mTORC2 complex, regulates ABLIM1 phosphorylation, influencing these cellular processes. This interaction suggests that targeting the Rictor-ABLIM1 axis could be a viable strategy to inhibit HCC progression (27). In contrast, in glioblastoma (GBM), ABLIM1 acts as a tumor suppressor, where its overexpression is associated with reduced tumor size and improved patient survival (28), implicating the diverse role of ABLIM1 in different cancer context. Our study showed that ABLIM1 was broadly downregulated in uterine tumor samples, indicating a possible protective role during the transformation of uterine smooth muscle cells. Although the precise molecular mechanisms remain to be elucidated, current evidence supports the clinical value of ABLIM1 as a putative tumor suppressor.

FHL5 is a member of the LIM-only protein family, which is known for its role in transcriptional regulation. FHL5 has recently been identified as a candidate gene in coronary artery disease and myocardial infarction, suggesting its involvement in vascular remodeling and disease risk through transcriptional regulation of downstream gene programs (29). Studies about the role of FHL5 in cancer are limited. In colorectal cancer, FHL1 has been reported to be a tumor suppressor gene by negatively regulating the Wnt/β-catenin signaling pathway (30). Our study showed the consistent downregulation of FHL5 in uterine tumor tissues, with significant association with poor prognosis. This suggests a potential involvement of FHL5 in regulating the tumor microenvironment or suppressing tumor cell proliferation, warranting further functional investigation.

MAP3K8, also known as TPL2 or COT, plays a significant role in various cellular processes, including inflammation, cell proliferation, and survival. Similar to ABLIM1, it has been reported to play dual role in cancer dependent on cancer type. High expressions of MAP3K8 in renal clear cell carcinoma and glioma have been reported to correlated with poor survival (31, 32). In hepatocellular carcinoma, MAP3K8 has been reported to promote tumor progression through regulating PD-L1^+^ macrophage infiltration, indicating its immune regulatory role (33). In contrast, in lung cancer and breast cancer, MAP3K8 has been reported to be a tumor suppressor, patients with high MAP3K8 expression showed better prognosis (34, 35). We also identified MAP3K8 as being stably downregulated in uterine tumors, with its reduced expression positively correlated with favorable prognosis. This expression profile supports a possible tumor-suppressive function of MAP3K8 in this context.

Although incorporating all available datasets into a unified statistical analysis could theoretically increase sample size, the substantial molecular heterogeneity across uterine sarcoma subtypes—especially the divergent transcriptomic profiles between ULMS and endometrial stromal sarcomas—may introduce systematic bias. Therefore, we restricted our primary analysis to four datasets with clearly defined sample types and matched normal controls, while using other datasets for independent validation. This strategy ensured both the reproducibility and biological validity of our findings. Despite the inherent heterogeneity among public datasets, the consistent expression trends of the identified molecular markers across independent cohorts strongly support their clinical potential as common biomarkers for uterine tumors.

In conclusion, this study systematically identified four core genes—ABLIM1, FHL5, MAP3K8, and TOP2A—that are consistently dysregulated in uterine smooth muscle tumors relative to normal tissue, through an integrated bioinformatic approach. These genes demonstrated robust and reproducible expression patterns across multiple independent datasets and were further validated at the protein level. Our findings not only shed light on potential molecular drivers of uterine tumorigenesis but also provide a molecular foundation for the development of more sensitive and specific tools for early diagnosis. Additionally, the four-gene signature exhibited clear prognostic value, indicating its potential utility in risk stratification models. Future functional validation and prospective clinical studies will be crucial to advancing the clinical translation of these biomarkers and opening new avenues for precision diagnosis and therapy in uterine tumors.

## Data Availability

The raw data supporting the conclusions of this article will be made available by the authors upon reasonable request, without undue reservation.
