# Supplementary material for: A four-gene signature identified by integrated transcriptomic analysis for differential diagnosis and prognosis of uterine smooth muscle tumors
Source: Front Oncol. 2025 Aug 4;15:1591875. doi: 10.3389/fonc.2025.1591875 (PMC12358259; doi:10.3389/fonc.2025.1591875)
Supplement: Supplementary file 1 [file Image1.pdf]

Figure S1. Genes identified by three complementary bioinformatics approaches (GEO2R, Limma and WGCNA)

GEO2R	Limma	WGCNA									
ABCA8	COL5A2	SLC46A3	ZNF160	ARL4A	ACSS3	NPR1	GIMAP4	EFEMP1	RRM2
ABLIM1	HOPX	JADE3	TP53	TSC22D3	SORBS3	FAXDC2	RASIP1	MYRIP	CCNB1
BHMT2	NREP	ZMAT3	ELN	NEDD9	CROCCP2	NR2F2	FZD4	ACSL5	HELLS
C1S	CDH2	NQO1	ENAH	EPHX1	ITGA7	PLN	TLR3	CLDN5	CKAP2
CITED2	CAPN6	RASL12	GOLGA6L9	PDGFC	EPPK1	C1R	WFS1	PDE2A	POLE2
CKS2	CEMIP	ANGPT2	EGR3	F8	KCNH2	OSR2	LTBP4	CD200	CDK1
DPT	IGSF3	MFGE8	SDC3	JAG2	PRKAR2B	DMPK	TSPAN12	KLF4	TRIP13
FHL5	EGFL6	MPPED2	ZNF432	AHNAK2	POLI	CX3CR1	RASSF9	NR3C2	ECT2
KIT	DCX	KAT2B	MICAL3	OSBPL1A	GEM	RASSF2	CRIP2	MMRN2	MCM2
KLF2	MTCL1	LANCL2	RAP1GAP2	C2CD2	FZD7	OGN	KLF2	KDR	NT5DC2
LIFR	IL17B	WDR19	CFHR1	PDZD2	IER2	JUN	FHL5	AQP1	NETO2
MAFB	MMP11	MYOM1	ANKRD28	HOXA10	PTPRF	FGF2	ADIRF	FBLN5	UBE2C
MAP3K8	NPTX2	ZNF395	CORO2B	PDLIM1	SOD3	PODXL	RUNDC3B	TIE1	CDC7
MS4A2	TUBB2B	FAM20B	TRMT11	PTPRO	MCAM	ID4	VASH2	SOX17	SHOX2
PAGE4	ADAM12	NAALAD2	ADAM9	MTUS1	RTN2	ADAMTS5	ATP6V0E2	GIMAP6	SAC3D1
SATB2	SHOX2	CD46	GNB5	ZNF423	VLDLR	REV3L	ESM1	PECAM1	SDC1
SDC1	PPFIA4	AGTR1	FBN1	DAPP1	STK38L	EMX2	COL4A1	EMCN	HMGB3
SPP1	COL11A1	ARHGEF6	FGF13	TRIM34	MGLL	PHYHIP	SPI1	KCNN3	TMSB15A
TFPI	TOP2A	HLA-DQA1	NEK7	HOPX	COL6A2	PLAT	SERPINE2	RNASE4	LMNB1
TOP2A	ZNF365	AP1S2	PDLIM2	DPYD	APOL1	CD34	FSCN1	LMO2	GMNN
TYMS	ABCA6	RGL1	HBG1	ELMO1	GALNT7	DYNC2LI1	PAK3	LPAR6	ZWINT
	RGS13	HCP5	HDAC9	SMAGP	PLK2	IGF1	AGO2	CBX7	PMAIP1
	SCN7A	ARGLU1	STAT3	HOXD4	HYAL2	MAOB	KCNK3	RAMP3	GINS1
	COL4A4	TOR1AIP1	ZNF302	RAMP2	ST5	STON1	ABCA1	FAM110D	ZNF365
	CRHBP	MYLIP	DMXL1	SERPING1	CTSH	ABCG2	ITGB2	SELP	MAFB
	IL20RA	RSBN1	ZCCHC2	MITF	GLUL	AOC3	RBBP5	PCNA	PAFAH1B3
	ASPA	ACACB	ARHGAP29	HPCAL1	IFIT1	FXYD1	MAP3K7CL	RMI1	TUBG1
	CYP4B1	SECISBP2L	RPS6KA5	ITIH5	HLA-DRB1	PPP1R15A	SRSF6	NID2	IFI30
	FMO2	HLA-DRA	ARHGAP5	TPST2	DUSP6	KLF6	ALPL	MAN2B1	COL11A1
	ARAP2	GPR37	INSR	SEMA3G	CD52	RASGRP3	PPP3R1	STMN1	KPNA2
	CORIN	CPEB3	DOCK4	ZNF516	AMIGO2	BIRC3	DOCK2	MFAP2	RACGAP1
	TLR3	PLA2G4C	STXBP1	HYAL1	PLAGL1	PLCE1	TGIF2	MAGED1	ZWILCH
	FHL5	VPS36	GCH1	LRP5	MAFF	TXNIP	PTH2R	TP53I3	COL5A2
	F10	RIMBP2	TBX3	HEG1	JUP	FGL2	INPP4B	MAGED4B	NPTX2
	LYVE1	MSLN	PDZRN3	LHX6	LGALS3	DACH1	RNASET2	BAX	NDC80
	MAP3K8	ELF1	JMJD1C	DPP6	HSD17B11	TFPI	MYH10	LOXL2	COL9A2
	ADAMTSL3	PLVAP	PXDC1	MALL	NACC2	EPAS1	FKBP14	COTL1	MCM7
	BHMT2	CX3CL1	CTSC	CTF1	BDKRB2	EMP1	SLC2A3	WASF1	RRAGD
	LIFR	NR5A2	PBX1	PDGFD	MXI1	IRAK3	ESF1	THEMIS2	ENC1
	GATA2	ALDH1A2	SYNE2	SACS	SORL1	TNFRSF11B	IDH2	MEST	HMOX1
	ABLIM3	ZSCAN31	HLA-DPB1	TUBA4A	ADD3	ANG	CDH2	FARP1	FZD2
	SYBU	CD93	SULT1A1	LOXL1	AR	MS4A2	TMEM97	PEG10	PGD
	PPL	HOXA5	PRKCZ	KLF10	C3	PHACTR2	SMS	COL4A2	OLFML2B
	TRPC4	NAV2	SSBP3	PPIP5K2	SYNGR2	DUSP1	PHGDH	FABP5	MTHFD2
	RERGL	SLC39A4	CCNG2	NEK4	AKR1C2	PTGIS	H2AFZ	FKBP10	TUBB2B
	OMD	GOLM1	ING3	PMEPA1	PIK3CA	TMEM47	VAV3	ELP5	CDKN2C
	EDNRB	LRRC59	NRGN	LBR	SMAD1	NUAK1	TNFAIP6	SLC43A3	SPP1
	KRT19	MAZ	GDE1	FADS2	TLE2	WBP4	TNFRSF21	HOMER1	MTCL1
	GPM6A	ERI2	HLA-DRB5	CAPRIN1	HSPB7	TSPAN2	RASA4	NPM3	NRAS
	KIT	ROBO1	LTBP3	NAMPT	PDGFA	SOX18	FILIP1L	ANXA6	MICB
	ADH1B	HLX	CLK4	TSPAN15	AMOTL2	RRAS	SGMS1	MX2	C1QL1
	FOSB	USP6	NRN1	RHOBTB1	KANK1	PDLIM3	RBMS3	NONO	HMGB2
	ABCA8	EHD3	CD74	PRPF39	ATF3	PDGFRL	GSN	BSG	FHOD3
	TPSAB1	PFKFB3	RNF19B	PLP2	RCAN2	FLI1	C1S	NPIPB3	
	KLF4	POMZP3	SNRPN	VASP	CHRDL1	SNAP23	CES1	NUSAP1	
	ZBTB16	MMP2	LTBP2	LTBP1	TJP2	NOS3	RYR3	TOP2A	

	DPT	MICALL1	LY75	AGL	PPP1R12B	FAM149A	ADRA2C	PRC1
	ABLIM1	TNFSF13	C1QTNF1	CHST2	GRK5	ABCA6	THBD	GGH
	CPA3	RBBP4	RCBTB1	SHC2	BCAM	PCOLCE2	SPRY1	MAD2L1
	ZFPM2	FAM114A1	EPS8L2	COL18A1	CLEC2B	NDRG2	TMEM204	PTTG1
	ATF3	VCAM1	ARID5A	PLTP	TPPP3	NFIB	PALMD	CCNB2
	IGFBP6	LGALS9	KLF7	FCGBP	CLU	MEOX2	ABLIM1	RAD51AP1
	ALDH1A1	SERINC5	CSF2RB	PSMB9	SPEG	CLDN1	THSD7A	CKS2
	RBP1	PER1	INPP5A	SLC16A1	CLIP1	MAP3K8	LRRC32	KIF11
	C1R	DOK5	NR3C1	TNFAIP8	ITGA6	CCL23	ID1	MELK
	CITED2	DDAH1	VEGFA	JUNB	PIK3R1	TJP1	PLCB4	TPX2
	C7	HERC2P2	PTPRE	EMP2	MRC1	KL	ERG	BUB1B
	DUSP1	GSTM2	TIMP3	CAND2	MYL9	FRZB	APOL3	EZH2
	APOD	GOLGA8N	UGP2	SPON2	CSDC2	SPON1	MMP23B	CENPU
	FOS	LUC7L3	LSR	LSP1	CELF2	RERGL	CLIC2	DTL
	HBB	MRC2	RHOB	LBH	GPRASP1	FMO2	EGFL7	TYMS
